# XEN Gel Stent in the management of primary open-angle glaucoma

**DOI:** 10.1007/s10633-020-09753-4

**Published:** 2020-02-12

**Authors:** Michał Post, Wojciech Lubiński, Dominik Śliwiak, Karolina Podborączyńska-Jodko, Maciej Mularczyk

**Affiliations:** 1 2nd Chair and Department of Ophthalmology, Pomeranian Medical University in Szczecin, Al. Powstańców Wlkp. 72, 70-111 Szczecin, Poland; 2 Chair and Department of Human and Clinical Anatomy, Pomeranian Medical University in Szczecin, Al. Powstańców Wlkp. 72, Szczecin, 70-111 Poland

**Keywords:** Glaucoma, Minimally invasive glaucoma surgery, MIGS, XEN, Pattern electroretinogram, PERG

## Abstract

**Purpose:**

To assess the efficacy and safety of the XEN Gel Stent in patients with primary open-angle glaucoma.

**Materials and methods:**

Twenty eyes of 17 patients (6 males, 11 females) with primary open-angle glaucoma were implanted with XEN Gel Stent. The following data were ascertained in each participant at baseline and at 1, 3, 6, 9 and 12 months following implanting procedure: intraocular pressure, number of anti-glaucoma medications, retinal sensitivity (PS 24/2 w/w), pattern electroretinogram (ISCEV standard), as well as the number of complications.

**Results:**

The mean intraocular pressure reduction in a 1-year follow-up was 18% (21.56 vs. 17.69 mmHg, *p* < 0.001). The mean number of anti-glaucoma medications was reduced from 3.2 to 1.6 (*p* = 0.001). The PERG parameters at baseline and at 12 months postoperatively included a stable amplitude of P50 (2.55 µV vs. 2.65 µV, *p* = 0.024) and N95 (3.45 µV vs. 3.38 µV, *p* = ns) waves. The delta N95 and delta P50 amplitudes remained stable over the follow-up period (*p* = ns). The mean deviation (MD) of PS 24/2 was − 6.54 dB vs. − 8.43 dB, *p* = ns, whereas the pattern standard deviation (PSD) was 6.18 dB vs. 6.91 dB, *p* = ns. Transient hypotony within the first postoperative week occurred in 18 eyes (90%), whereas hyphema occurred in two eyes (10%). Needle revision of a filtration bleb was performed in five eyes (25%).

**Conclusions:**

The XEN Gel Stent enables significant reduction in intraocular pressure with very low complication rates. It ensures a stabilization of the retinal function as established with the PERG.

## Introduction

Glaucoma is an optic nerve pathology, which affects approximately 60 million people worldwide [1]. The main treatment goal is to reduce intraocular pressure (IOP) using medications, laser or surgical procedures. Introduced in 1968, trabeculectomy is currently considered a gold standard in glaucoma surgery. Although effective, it is associated with a risk of serious complications such as hypotony, bleb leaks, cataract and others [2–4]. The main cause of trabeculectomy failure is conjunctival/tenon fibrosis, which occurs in 25–30% of patients [5]. Furthermore, blebitis and bleb-related endophthalmitis may occur, with a cumulated risk of 1.1% in a 5-year follow-up [4–6].

Therefore, new, safer surgical methods of reducing the IOP are still being sought. In recent years, the minimally invasive glaucoma surgery (MIGS) has developed dynamically. Although it has not been precisely defined, it is assumed that MIGS includes all procedures which improve the outflow of the aqueous humour offering fast recovery and low complication rates. These are usually performed via an *ab interno* approach, that is, without the need of conjunctival incision. Out of all MIGS procedures, only XEN Glaucoma Microstent (XEN-GGM, Allergan Plc, Parisppanny, NJ, USA) improves aqueous outflow by creating the subconjunctival outflow pathway. All other MIGS procedures improve the outflow via either Schlemm’s canal or suprachoroidal pathway [7, 8]. The XEN-GGM is a trans-scleral gel stent implanted through the clear corneal incision. It provides drainage from the anterior chamber to the subconjunctival space. Some studies show that the efficacy of the XEN-GGM is comparable to the one of trabeculectomy with the former making it possible to avoid conjunctival incision and being associated with lower complication rates [7–9]. Currently, XEN-GGM is recommended as a stand-alone procedure or combined glaucoma and cataract surgery in patients with mild-to-moderate glaucoma and unstable IOP despite medical therapy [10]. XEN-GGM received the CE Mark in December 2015 and FDA approval in November 2016. Since then, several studies have been carried out to assess the efficacy of the XEN-GGM. Most of them (including our study) focus on IOP reduction, the number of anti-glaucoma medications and complications [7–10]. However, unlike other published studies, ours is the first to determine the transient pattern ERG trend after XEN-GGM execution.

## Methods

### Patients

Twenty pseudophakic eyes of 17 patients (6 males, 11 females) with primary open-angle glaucoma were implanted with XEN Gel Stent. The mean age was 69.85 ± 4.59 years. Prior to the procedure, all eyes had been treated conservatively with anti-glaucoma topical medications and argon laser trabeculoplasty. In all cases, the glaucomatous disease showed signs of progression despite maximal topical therapy.

The following data were ascertained in each participant at baseline and at 1, 3, 6, 9 and 12 months following implanting procedure: IOP (Pascal tonometry), number of anti-glaucoma medications, number of complications and the best corrected distance visual acuity (BCDVA). Retinal sensitivity was measured by standard static perimetry (SITA 24-2 white on white threshold, Humphrey Visual Field Analyser), and the mean deviation (MD) and pattern standard deviation (PSD) were analysed. The transient PERG as per ISCEV standard (2013) [11] was performed at each follow-up visit. Patients with ocular (other than glaucoma) and systemic diseases with known influence on PERG recording were excluded from the research.

All procedures performed in studies involving human participants were in accordance with the ethical standards of the Institutional Research Committee and with the 1964 Declaration of Helsinki and its later amendments or comparable ethical standards. Informed consent was obtained from all enrolled study participants.

### XEN glaucoma microstent

XEN-GGM is a hydrophilic tube composed of a gelatine derived from porcine dermis and cross-linked with glutaraldehyde. Its design and gelatine content make it 100 times more flexible than silicone while not causing foreign body reaction. As a result, the local conjunctival reaction to the implant seems minimal [9]. XEN-GGM is 6-mm long. Its outer and inner diameters are 220 µm and 45 µm, respectively [9]. In keeping with the Hagen–Poiseuille equation, the stent diameter generates > 7.6 mmHg outflow, whereas the physiological aqueous humour production is 2.5 µl/min [12]. Therefore, it prevents persistent postoperative hypotony through a non-valve mechanism.

All XEN-GGM implanting procedures were carried out under a local anaesthesia. The insertion site was marked within the superior nasal conjunctival quadrant at 3 × 3 mm from the limbus, and 0.1 ml of 0.02% mitomycin C was applied to the site. Then, a 1.5–1.7-mm-wide clear corneal incision was created at the 7 o’clock position with a 1.2-mm-wide additional incision at the 11 o’clock position in right eyes. The incision locations were reversed if the left eye was treated. A cohesive viscoelastic, 1% lidocaine and a miotic agent (Carbachol) were administered to the anterior chamber. The needle of the sterile XEN injector preloaded with the XEN Gel Stent was advanced through the clear corneal incision via the *ab interno* approach, and the stent was implanted into the previously marked subconjunctival space. After a correct position of the XEN-GGM was ensured, viscoelastic was irrigated and aspirated from the anterior chamber, the wound was hydrated, and 0.1 mL of cefuroxime was administered into the anterior chamber. After the procedure, all anti-glaucoma medications were discontinued in the treated eye. During the postoperative period, all patients were administered dexamethasone (DexaFree, Thea) five times a day for a total of 5 weeks with a reduction by 1 drop per week and floxacillin (Floxal, Bausch) four times a day for 1 week.

### Pattern electroretinogram

**Fig. 1:**
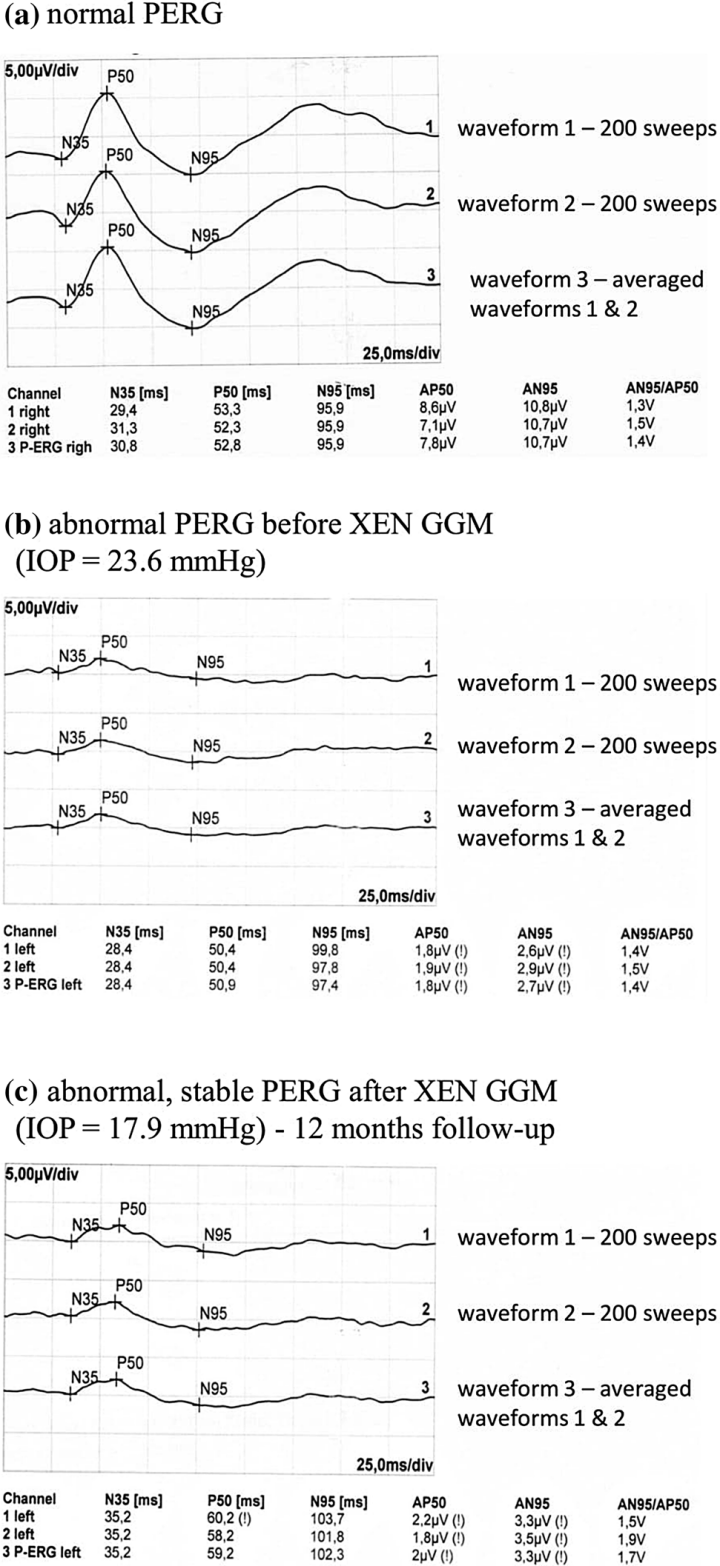
Example of PERG amplitude of P50 and N95 waves: **a** normal **b** abnormal before XEN-GGM implantation **c** abnormal after XEN-GGM implantation

The transient PERG was recorded with the RetiPort system (Roland Consult Instr.) according to the ISCEV standards [11] from undilated eyes. Appropriate refractive error correction in relation to the eye-screen distance was ensured. Monocular stimulation was used. The following parameters of the recording system were preset: filters were set at 1–100 Hz, notch filters were off, and amplifier sensitivity was set at 20 μV/div. The artefact rejection threshold was set at 95% with the amplifier range of ± 100 μV. The sweep time was set at 250 ms with the time base set at 25 ms/div and the mean number of sweeps set at 200. Two waveforms were recorded (Fig. 1a waveforms 1, 2), then analysed and averaged offline (Fig. 1a waveform 3). The automatic cursor placement was corrected manually if necessary. The values of all parameters were compared to the laboratory age-matched normative database: amplitude P50 3.2–11.3 µV, implicit time P50 46.5–59.2 ms, amplitude N95 4.8–15.7 µV, implicit time N95 87.2–117.9 ms, the N95/P50 ratio 1.13–1.69. At baseline, 2/20 eyes were within normal limits, 18/20 eyes had pathological records.

The parameters of the pattern stimulation were the following: 21″ CRT monitor with a frame rate equal to 75 fps was used; black-and-white contrast-reversing checkerboard (30° field of view (FOV)) was presented to the patient, with a check size equals to 1°2′; and the temporal frequency was 2.3 Hz. The electrodes placed were the following: the DTL electrode was an active electrode, the gold disc electrode was placed as a ground electrode over the forehead (Fpz) and another gold disc electrode was placed as a surface reference electrode on the skin near the ipsilateral outer canthus of the eye.

### Statistical analysis

The descriptive statistics including means, standard deviations and ranges of eight assessed parameters (IOP, AP50, AN95, AP95, PT P50, MD, PSD, numbers of medications) were calculated for 6 time points (baseline—before treatment, and at months 1, 3, 6, 9 and 12 postoperatively). The normality of distribution was verified using the Shapiro–Wilk test. The obtained parameters were analysed using Friedman’s ANOVA with post hoc tests. The correlation between variables was analysed using linear Pearson’s coefficient. The results were considered significant with *p* < 0.05. All data were analysed using STATISTICA 12.5 software.

## Results

Figure 1 presents example of PERG amplitude of P50 and N95 waves in healthy subject (Fig. 1a) and glaucoma patient before (IOP = 23.6 mmHg, Fig. 1b) and after (IOP = 17.9 mmHg, Fig. 1c) XEN-GGM implantation.

### Intraocular pressure and number of IOP-lowering medications

The IOP was significantly reduced (18%) at 12 months as compared to baseline (21.56 ± 2.29 mmHg vs. 17.69 ± 2.11 mmHg, *p* < 0.001, Table 1). In five patients (25%), the IOP dropped by > 25%, while the number of anti-glaucoma medications decreased from 3 to 1. There was no hypotony after 12 months, and no patient required an intervention due to low IOP. The number of anti-glaucoma medications was significantly reduced at 12 months as compared to baseline (3.20 ± 0.77 vs. 1.60 ± 0.99, *p* < 0.001, Table 1).

**Table 1:** Intraocular pressure (IOP) and the number of IOP-lowering medications before and after treatment. *p* < 0.001 for both comparisons (Friedman’s ANOVA)

		IOP				Number of IOP-lowering medications			
	*n*	Mean	SD	Min	Max	Mean	SD	Min	Max
Baseline	20	21.56	2.29	18.00	25.10	3.20	0.77	2	4
1 month	20	15.76	2.02	11.50	19.00	0.15	0.37	0	1
3 months	20	15.43	2.18	11.50	18.00	0.35	0.59	0	2
6 months	20	16.21	1.75	13.20	19.00	0.80	0.62	0	2
9 months	20	17.53	2.19	12.80	22.00	1.40	0.82	0	3
12 months	20	17.69	2.11	13.00	22.30	1.60	0.99	0	3

### Pattern electroretinogram

**Fig. 2:**
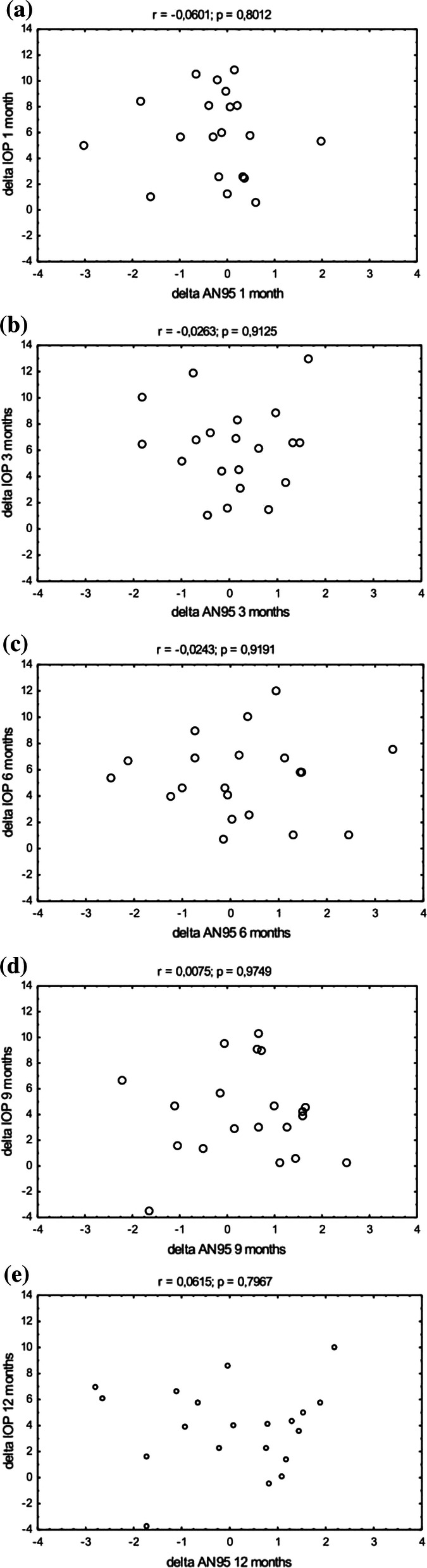
Scatterplots of delta IOP as a function of delta PERG AN95 in all patients (*n* = 20). **a** 1 month after surgery, **b** 3 months after surgery, **c** 6 months after surgery, **d** 9 months after surgery, **e** 12 months after surgery

**Fig. 3:**
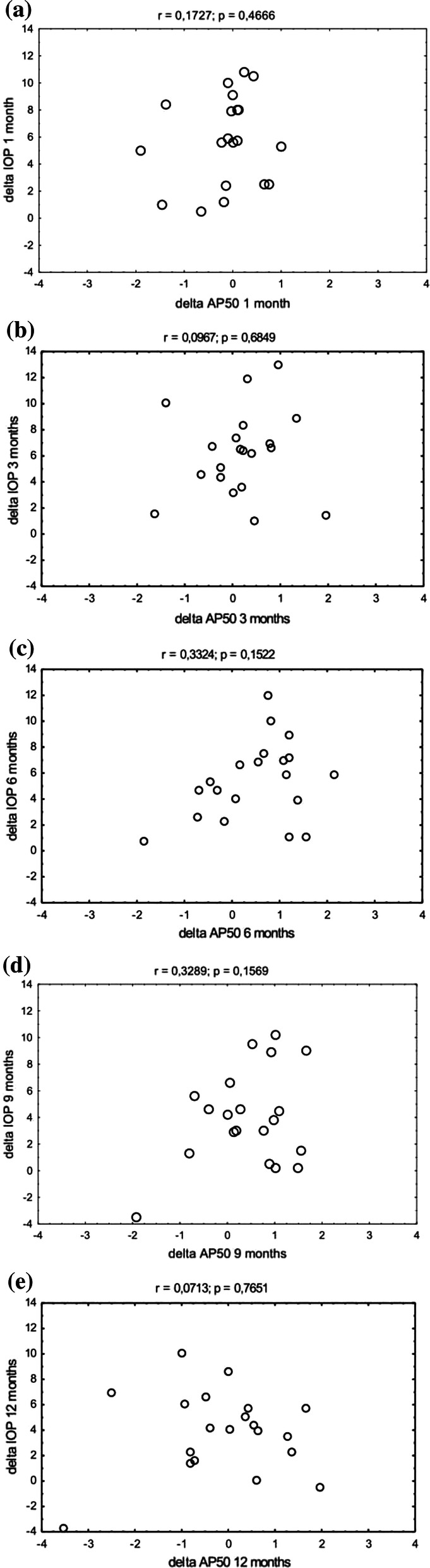
Scatterplots of delta IOP as a function of delta PERG P50 in all patients (*n* = 20). **a** 1 month after surgery, **b** 3 months after surgery, **c** 6 months after surgery, **d** 9 months after surgery, **e** 12 months after surgery

The amplitudes of the P50 and N95 waves remained stable over the follow-up period (Table 2). The N95 was 3.45 ± 1.61 µV and 3.38 ± 1.69 µV at baseline and at 12 months, respectively (*p* = ns). Figure 2 shows that delta IOP and delta N95 were not correlated over the follow-up period (Fig. 2). The P50 was 2.55 ± 1.23 µV and 2.65 ± 1.03 µV at baseline and at 12 months, respectively (*p* = ns). Figure 3 shows that delta IOP and delta P50 were not correlated over the follow-up period (Fig. 3). The P50/N95 ratio was 1.36 ± 0.47 and 1.26 ± 0.44 at baseline and at 12 months, respectively (*p* = ns). The implicit time of the P50 wave remained stable over the follow-up period (54.01 ± 8.15 ms vs. 57.37 ± 4.73 ms at baseline and at 12 months, respectively, *p* = ns,). In a subset of patients with IOP reduction by > 25% (*n* = 5), there was no significant change of PERG parameters. The N95 and P50 amplitudes and the P50 implicit time were 3.17 ± 1.19 vs. 3.44 ± 1.44, 2.35 ± 0.89 vs. 2.8 ± 1.1 and 53.26 ± 4.87 vs. 51.74 ± 5.52, respectively (*p* = ns for all comparisons).

**Table 2:** P50 and N95 amplitudes as well as the P50 implicit time before and after treatment

	*n*	P50 amplitude (µV)				P50 implicit time (ms)				N95 amplitude (µV)			
		Mean	SD	Min	Max	Mean	SD	Min	Max	Mean	SD	Min	Max
Baseline	20	2.55	1.23	0.71	4.79	54.01	8.15	44.00	78.30	3.45	1.61	0.49	6.23
1 month	20	2.68	1.43	0.89	5.88	56.91	7.19	45.00	79.30	3.69	1.99	0.59	7.70
3 months	20	2.37	1.05	0.61	4.38	56.53	7.34	47.00	79.30	3.36	1.41	0.75	6.20
6 months	20	2.04	0.85	0.53	3.23	56.36	6.76	47.90	75.30	3.22	1.20	0.95	5.51
9 months	20	2.10	0.78	0.59	3.40	57.01	6.29	46.00	74.40	3.02	1.22	0.42	5.30
12 months	20	2.65	1.03	1.20	4.40	57.37	4.73	48.90	68.00	3.38	1.69	0.66	6.90

### Visual field (VF)

**Fig. 4:**
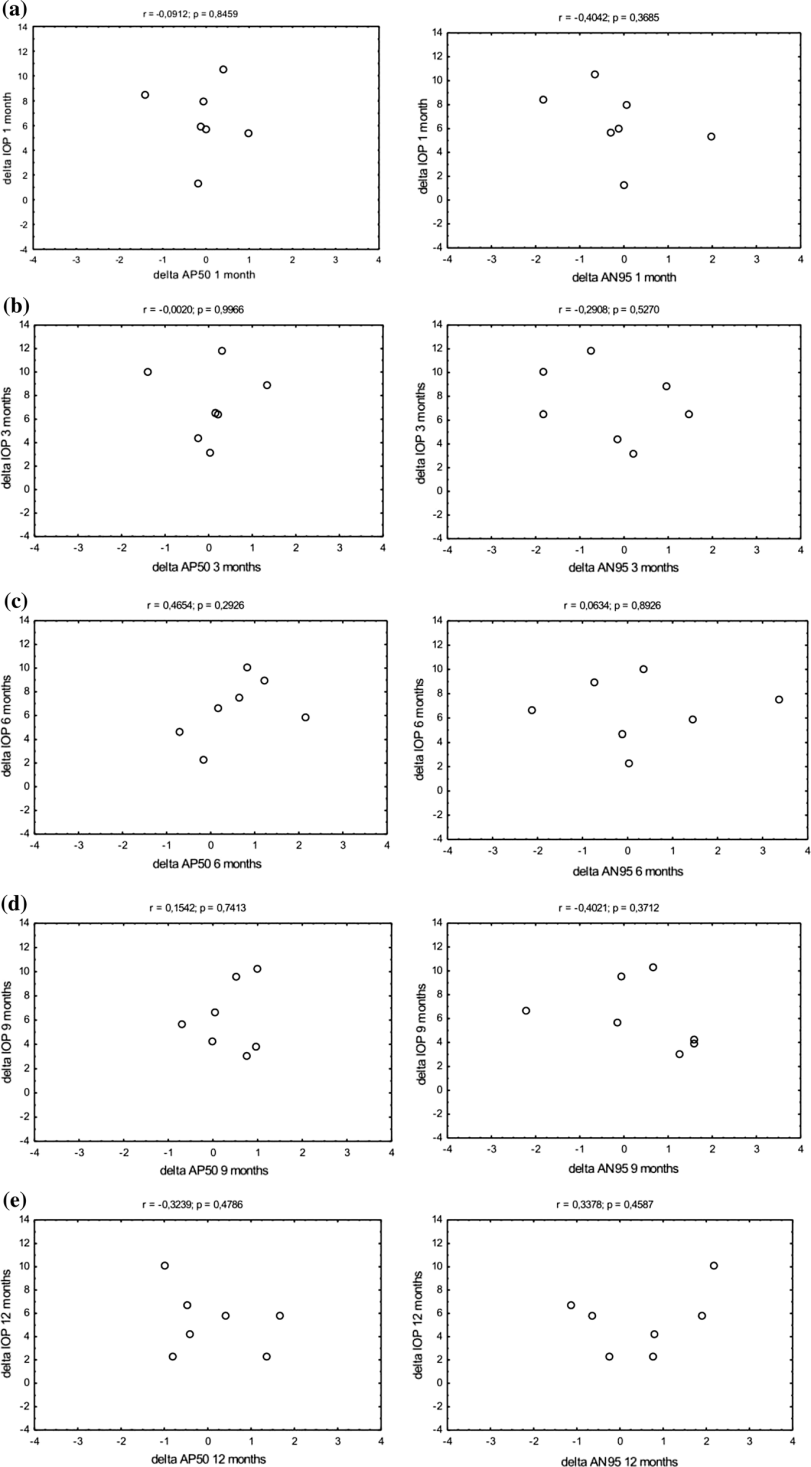
Scatterplots of delta IOP as a function of PERG AP50 (left column), PERG AN95 (right column) in patients with deterioration of MD in visual field (*n* = 7). **a** 1 month after surgery, **b** 3 months after surgery, **c** 6 months after surgery, **d** 9 months after surgery, **e** 12 months after surgery

The mean deviation (Humphrey perimeter) and pattern standard deviation at baseline were − 6.54 ± 3.30 dB and 6.18 ± 3.65 dB, respectively. (Table 3). The mean deviation and pattern standard deviation at month 12 postoperatively were − 8.43 ± 5.06 dB and 6.91 dB ± 3.42 dB, respectively. There was no significant change in the retinal sensitivity over 12 months. Figure 4 shows results in a subset of patients with deterioration of MD at 12 months (*n* = 7). In a subset of patients with deterioration of MD at 12 months, delta IOP was not correlated with neither AP50 nor AN95 over the follow-up period (Fig. 4).

**Table 3:** Characteristics of the mean deviation (MD) and pattern standard deviation (PSD) before and after treatment

	*n*	Mean deviation				Pattern standard deviation			
		Mean	SD	Min	Max	Mean	SD	Min	Max
Baseline	20	− 6.54	3.30	− 1.25	− 11.81	6.18	3.65	1.52	12.47
1 month	20	− 6.62	4.73	− 0.97	− 20.03	5.90	3.31	1.84	11.54
3 months	20	− 6.60	4.99	− 0.48	− 22.13	6.13	3.44	1.34	12.30
6 months	20	− 7.41	4.85	− 2.17	− 21.19	6.31	3.51	2.12	13.25
9 months	20	− 8.20	5.29	− 1.6	− 21.73	6.47	3.69	1.74	12.52
12 months	20	− 8.43	5.06	− 0.83	− 22.01	6.91	3.42	1.85	11.19

### Complications

The BCDVA remained stable over the 1-year follow-up and none of the patients lost > 2 lines (Snellen). Transient hypotony within the first postoperative week occurred in 18 eyes (90%), whereas hyphema occurred in two eyes (10%). Needle revision of a filtration bleb was performed in five eyes (25%). There were no cases of inflammation, anterior chamber shallowing, choroidal detachment, stent exposure or damage.

## Discussion

We observed 18% IOP reduction over a 1-year follow-up (21.56 vs. 17.69 mmHg, *p* < 0.001). This result is comparable to those of other studies with similar follow-up duration, where the IOP decreased by 25–56% to the mean level of 13–16 mmHg [10, 13–19]. Our results were obtained in pseudophakic eyes only. It is unclear whether a stand-alone procedure is more effective than phacoemulsification combined with XEN-GGM implantation. Hengerer et al. stated that this difference is non-significant [19]. Widder et al., on the other hand, observed higher efficacy of XEN-GGM in pseudophakic eyes (73%) as compared to phakic eyes (53%) or patients undergoing combined surgery (55%) [13]. In the study by Mansouri et al., the IOP reduction > 20% was achieved in 81% of patients after a stand-alone procedure vs. 56.1% of patients after combined surgery. However, the patients undergoing a stand-alone stent implanting procedure had a higher IOP at baseline.

In our study, the number of IOP-lowering medications was reduced by 50% (3.2 ± 0.77 vs. 1.6 ± 0.99, *p* < 0.001), which is in keeping with the results reported by Grover et al. (3.5 ± 1 vs. 1.7 ± 1.5, *n* = 65) [17]. In different studies of XEN-GGM, this reduction ranged between 50% and 100% [10, 13–15, 17–19]. In our study, needling was performed in five eyes (25%). This percentage is lower than in other studies with a 12-month follow-up where the mean needling rate was 32% (28–43%) [10, 13, 15–19]. In our opinion, our outcomes, slightly worse than those, may be explained by the learning curve and our more conservative approach during the postoperative period (higher use of anti-glaucoma topical medications, fewer revisions/ needling procedures). In line with this reasoning, Marques et al. found that having performed six procedures, a given surgeon had a lower risk of complications and the procedure duration was reduced to approximately 9 min [20]. Considering these findings, about a third of all observed patients underwent the procedure during the surgeon’s initial learning curve. Since XEN-GGM has only been available in Europe since 2015 and in the USA since 2016, there have been only a few studies with a longer follow-up. The only study with a 4-year follow-up reported a 40% IOP reduction (22.5 ± 4.2 vs. 13.4 ± 3.1, *n* = 34, *p* < 0.001), and a 50% reduction in the number of anti-glaucoma medications (2.4  ± 1.3 vs. 1.2  ± 1.3, *n* = 34, *p* < 0.001) [21]. Just like in our study, the results were affected by the surgeon’s learning curve. Significant differences, though, were the fact that Lenzhofer et al. did not use antimetabolites and performed both a stand-alone implantation (55%) and stenting combined with phacoemulsification (45%).

In our study, hypotonia-associated hyphema, which occurred in 10% of eyes, was transient and self-limiting. Similar studies also reported low rates of hyphema (0.7–5.6%), transient hypotonia (< 6 mmHg, < 1 month, 0.7–24.6%), anterior chamber shallowing (0.8–9.2%), choroidal detachment or choroidal folds (0.5–15%) and a positive Seidel test (1.4–9.0%) [10, 13–19]. In our study, there was no case of visual acuity loss of > 2 lines in a 12-month follow-up. In a study by Mansouri et al. [17], this occurred in 2% of patients in a 1-year follow-up, whereas in the study by Grover, such vision deterioration was confirmed in 6% of patients in a 1-year follow-up [16] and in 9% of patients in a 4-year follow-up [21]. On the other hand, 34% of patients after trabeculectomy had a visual acuity loss of > 2 lines at 3 years [28, 29]. In our study, the retinal sensitivity parameters assessed in a static perimetry (MD, PSD) remained stable over the follow-up period, which is in line with the study by Lenzhofera et al. with a 4-year follow-up [21]. It provides additional evidence that XEN-GGM enables IOP reduction and the number of anti-glaucoma medications preventing glaucoma progression.

The PERG recording predominantly reflects the activity of the inner retinal layers (mainly ganglion cells), with only minimum activity of the outer layers [22–24]. The parameters analysed in the PERG recording include the N95 amplitude, the P50 amplitude and the P50 implicit time [22–24]. The N95 is considered the most specific for glaucoma, as it is generated exclusively by the retinal ganglion cells (RGCs), whereas the P50 is formed as a result of a joint contribution of RGCs (70%), cones and cone bipolar cells (30%) [25, 26]. The PERG parameters reflect both retinal ganglion cell death and dysfunction of viable RGCs [27]. Function of RGCs may be restored to some degree by reducing the IOP; however, level of recovery seems to depend on many factors. Firstly to have significant improvement, the PERG must be abnormal. In studies of ocular hypertension, eyes with normal baseline PERG and treated with beta-blockers showed only slight PERG amplitude improvement of 10–20% [28–30]. Moreover normal subjects treated with acetazolamide showed no significant PERG changes, despite 30% IOP reduction [27]. However, in another study patients with severe PERG reduction, elevated IOP (32–56 mmHg), but normal visual field had significant PERG restoration (up to 200%) after IOP reductions by approximately 30% with acetazolamide treatment [31]. Interestingly, PERG fluctuations after IOP reduction seem to depend on glaucoma type/baseline IOP level. In normal-tension glaucoma (NTG), PERG improvements, compared to chronic open-angle glaucoma (COAG) with elevated IOP, are associated with smaller IOP reductions [27]. Possible explanation might be greater susceptibility of RGCs of NTG eyes to IOP elevation than those of COAG eyes. Pattern ERG improvement due to IOP lowering may occur in COAG, NTG and ocular hypertension; thus, PERG might be a tool for treatment effect monitoring. In the study by Karaskiewicz et. al., a 31% IOP reduction was associated with the P50 and N95 amplitude increase by 28% and 38%, respectively. The sensitivity was 75% and 79% for the P50 and N95, respectively [24]. Similarly Ventura and Porciatti conclude that PERG improvement with IOP lowering occurs in both high- and low-tension glaucoma eyes and is more expressed in eyes with mild VF defects [27]. In an experimental study in an animal model, the IOP reduction of 38% was associated with the increase in PERG wave amplitudes by 83% [32]. It is worth noting that in the above-mentioned studies, a significant improvement of RGCs function in PERG was associated with a reduction in IOP by 31% [24], 30% [31] and 38% [32]. In our study, the average decrease in IOP was only 18%. It is therefore possible that this reduction was too low to improve the RGCs function and PERG recordings. It should be also considered that the PERG was stable during follow-up because it was not sensitive enough to detect any ganglion cell function change in the eyes that were studied. The PERG tests carried out in this study (transient response) may be less sensitive in detecting ganglion cell dysfunction, compared to the steady-state response described by Ventura et al. [27]. An additional support for this thesis is the research by Bach et al. in which it was stated that for glaucoma studies, faster stimulation (steady state) is more efficacious than slower stimulation (transient) [33].

To date, no study has been published to discuss electrophysiological retinal findings in patients after MIGS (including those after XEN-GGM implantation). Our study is the first to analyse the changes in PERG parameters in patients after XEN-GGM implantation. In a subset of patients with IOP reduction by > 25%, there was a trend towards an improved ganglion cell function; the N95 and P50 amplitudes improved, and the P50 implicit time shortened. The difference was not significant, though, which can be attributable to a very small sample size (*n* = 5). There was no significant reduction in the P50 and N95 amplitudes and no significant deterioration of MD over 12 months in the entire sample (*n* = 20). This may indicate the therapeutic efficacy of XEN-GGM. Over the follow-up period both in whole group (*n* = 20) and in subgroup of patients with VF deterioration (*n* = 7), delta IOP was not correlated neither delta N95 nor delta P50 (Fig. 2-4). At the same time, there was no increase in the amplitude of P50 and N95 waves in the entire sample (*n* = 20). This result could be somewhat expected, as with abnormal PERG recording concomitant with visual field defect (early/moderate VF glaucomatous loss: MD − 6.54 ± 3.30 dB, PSD 6.18 ± 3.65 dB) at baseline, changes to PERG parameters occur not only as a result of impaired RGC function, but also due to RGC loss [34]. Similarly, many PERG studies indicate that greater PERG improvement after treatment is associated with normal or early altered VF, while less PERG recovery occurs in eyes with severe VF defects [27, 35, 36]. This may explain why patients with advanced glaucoma show poor PERG recovery or did not show recovery at all after glaucoma laser treatment [35] or trabeculectomy [36]. A possible explanation for the interaction between the extent of VF loss and PERG recovery is that in eyes with intact VFs, a larger number of RGCs is still viable, as compared with advanced states of glaucoma [27, 35, 36]. In other words, in patients with early VF impairment, there is a greater probability that retinal ganglion cell function can be at least partially restored after IOP reduction.

In our study, PERG parameters remained stable over the follow-up period. In our opinion, there are three possible explanations for this observation. First one is that IOP reduction after XEN-GGM implantation was too low to improve the RGCs function and PERG recordings. Secondly, PERG could be just not sensible enough to detect any ganglion cell function change in evaluated eyes Third possible explanation is based on fact that the PERG parameters reflect both retinal ganglion cell death and dysfunction of viable RGCs. It is possible that in the analysed group, RGC loss was the dominant factor for pathological PERG records and as a consequence, PERG did not improve after lowering the IOP.
